# Epigenetic Mechanisms in Sepsis-Induced Cardiomyopathy: From Pathophysiology to Therapeutic Targets

**DOI:** 10.3390/ijms27156767

**Published:** 2026-07-28

**Authors:** Chen Jiang, Zhanfei Li, Xijie Dong, Yuchang Wang, Xiaoxuan Zhong, Kan Wang

**Affiliations:** 1 Department of Nutrition, Tongji Hospital, Tongji Medical College, Huazhong University of Science and Technology, 1095 Jiefang Avenue, Wuhan 430030, China; 2 Trauma Center/Department of Emergency and Traumatic Surgery, Tongji Hospital of Tongji Medical College, Huazhong University of Science and Technology, 1095 Jiefang Avenue, Wuhan 430030, China; 3 Department of Cardiovascular Surgery, Tongji Hospital, Tongji Medical College, Huazhong University of Science and Technology, 1095 Jiefang Avenue, Wuhan 430030, China

**Keywords:** sepsis-induced cardiomyopathy, epigenetic regulation, DNA methylation, histone modifications, non-coding RNAs

## Abstract

Sepsis-induced cardiomyopathy (SICM) is a frequent and clinically important complication of sepsis, characterized by acute and often reversible myocardial dysfunction and associated with poor outcomes in critically ill patients. Despite growing recognition of its clinical significance, effective mechanism-based therapies remain unavailable, largely because SICM is highly heterogeneous and driven by a complex interplay of inflammatory activation, endothelial and microcirculatory dysfunction, oxidative stress, mitochondrial injury, calcium-handling abnormalities, metabolic reprogramming, and dynamic cardiomyocyte adaptation. In recent years, increasing attention has been directed toward epigenetic regulation, as it provides a mechanistic framework linking septic stress to sustained transcriptional and post-transcriptional remodeling in the myocardium and its immune microenvironment. Numerous studies have therefore investigated epigenetic mechanisms in SICM, including DNA methylation, histone modifications, and non-coding RNA-mediated regulation. This review summarizes recent advances in the understanding of epigenetic mechanisms underlying SICM, with emphasis on their roles in inflammation, mitochondrial dysfunction, immune–cardiac crosstalk, and cardiomyocyte injury. We further discuss the translational relevance of epigenetic remodeling and highlight the emerging therapeutic potential of epigenetically targeted interventions. Collectively, these findings provide important mechanistic insights into SICM and may support the development of biomarker-guided stratification and more precise therapeutic strategies for septic myocardial dysfunction.

## 1. Introduction

Sepsis is a life-threatening syndrome caused by a dysregulated host response to infection and remains a major cause of morbidity and mortality worldwide [1,2]. Cardiovascular dysfunction is one of the central manifestations of sepsis and is closely associated with hemodynamic instability, tissue hypoperfusion, and poor prognosis [3,4]. Within this context, sepsis-induced cardiomyopathy (SICM) has attracted increasing attention as an acute and often reversible form of myocardial dysfunction occurring during sepsis, although its definition and diagnostic criteria remain incompletely unified [5,6,7]. Current clinical understanding generally recognizes SICM as new-onset myocardial dysfunction unrelated to coronary artery disease, involving left ventricular and/or right ventricular systolic and/or diastolic impairment, yet the extent to which isolated diastolic dysfunction or isolated right ventricular dysfunction should be incorporated into the definition remains debated [8]. Reported prevalence varies widely across studies because of differences in patient populations, imaging modalities, diagnostic thresholds, timing of assessment, and loading conditions during resuscitation [9,10].

Importantly, SICM is increasingly recognized as a heterogeneous and dynamic syndrome rather than a uniform cardiac disorder. Left ventricular systolic dysfunction, preserved ejection fraction with impaired myocardial strain, isolated diastolic dysfunction, right ventricular dysfunction, and biventricular impairment may represent distinct clinical phenotypes with different degrees of reversibility and clinical outcomes [2]. This phenotypic diversity is accompanied by substantial mechanistic heterogeneity, involving inflammatory mediator release, impaired adrenergic responsiveness, calcium-handling abnormalities, mitochondrial dysfunction, oxidative and nitrosative stress, coronary microvascular perturbation, endothelial dysfunction, and metabolic reprogramming or myocardial hibernation [11]. More recent mechanistic work at the single-nucleus level further suggests that septic cardiac dysfunction may involve cardiomyocyte subtype conversion from a contractile state to an injury-responsive state, a process regulated in part by estrogen-related receptor γ (ERRγ), thereby adding a new cellular dimension to the pathophysiological complexity of SICM [12,13,14]. However, the molecular mechanisms underlying different SICM phenotypes and their potential relevance for patient stratification remain incompletely understood.

Given the marked clinical and biological heterogeneity of SICM, identifying molecular mechanisms that contribute to phenotype-specific responses has become increasingly important. Epigenetic regulation provides a potential framework linking septic stress to persistent transcriptional and phenotypic remodeling in cardiomyocytes and the cardiac immune microenvironment. Alterations in DNA methylation, histone modifications, and non-coding RNA expression may influence inflammatory activation, mitochondrial adaptation, oxidative stress responses, metabolic remodeling, and cardiomyocyte survival programs. Therefore, phenotype-associated epigenetic signatures may represent promising biomarkers for SICM classification and therapeutic stratification. However, current evidence directly connecting specific epigenetic alterations with individual SICM phenotypes remains limited, highlighting an important area for future investigation. Epigenetics refers to reversible and heritable changes in gene expression that occur without alteration of the DNA sequence, and its major mechanisms include DNA methylation, histone modifications, and non-coding RNA-mediated regulation; recent studies have also extended this framework to RNA modifications [15,16]. In cardiovascular diseases, epigenetic regulation has emerged as an important mechanism linking environmental stress, inflammation, metabolism, and altered transcriptional programs to disease initiation and progression [17]. DNA methylation typically modulates transcription through CpG-associated chromatin changes, histone modifications regulate chromatin accessibility and effector recruitment, and non-coding RNAs, including microRNAs, long non-coding RNAs, and circular RNAs, exert multilayered control over transcriptional and post-transcriptional networks [18]. RNA modifications have also emerged as a novel frontier connecting cellular metabolism with gene regulation [19,20]. These mechanisms do not operate independently; rather, they form an interconnected regulatory network capable of translating inflammatory and metabolic stress into sustained phenotypic remodeling. In the setting of sepsis, where immune dysregulation, oxidative injury, mitochondrial stress, and metabolic adaptation are prominent, such a framework is particularly relevant and may help explain how transient systemic infection triggers dynamic yet potentially reversible myocardial dysfunction [16].

Given the substantial clinical burden of sepsis, the incomplete understanding of SICM, and the lack of specific mechanism-based therapies, it is necessary to systematically summarize the emerging evidence on epigenetic regulation in septic myocardial injury. Although supportive care, including source control, fluid resuscitation, vasopressor therapy, and selected inotropic support, remains the current cornerstone of treatment, no targeted therapy for SICM has yet been established, largely because its pathophysiology is multifactorial and temporally heterogeneous [13]. Recent SICM literature also suggests that future progress will likely depend on better phenotyping, biomarker profiling, translational research, and more precise identification of targetable molecular pathways. In this review, we discuss the current understanding of SICM pathophysiology and then focus on the major epigenetic mechanisms potentially involved in this process, including DNA methylation, histone modifications, and non-coding RNAs, with emphasis on how these pathways intersect with inflammation, oxidative stress, mitochondrial dysfunction, calcium dysregulation, and immune-metabolic disturbance. We further summarize the potential translational significance of these findings and discuss the opportunities and challenges of epigenetic-based therapeutic strategies for SICM.

## 2. Pathophysiology of Sepsis-Induced Cardiomyopathy

### 2.1. Definition and Clinical Manifestations of Sepsis-Induced Cardiomyopathy

Sepsis-induced cardiomyopathy (SICM) is generally regarded as an acute, sepsis-associated, non-ischemic cardiac dysfunction characterized by impaired left ventricular systolic and/or diastolic function, right ventricular dysfunction, or biventricular involvement, although no universally accepted diagnostic criteria have yet been established [12,13]. Contemporary reviews emphasize that SICM should not be equated simply with a reduced left ventricular ejection fraction, because both ejection fraction and cardiac output are highly dependent on loading conditions and may therefore fail to reflect intrinsic myocardial contractility during septic shock [21]. Clinically, SICM often presents with hemodynamic instability, reduced cardiac efficiency, impaired ventricular relaxation, and abnormal ventriculo-arterial coupling in the setting of sepsis or septic shock [4]. Earlier descriptions emphasized reversible left ventricular dilatation and depressed ejection fraction with recovery within 7–10 days in survivors, whereas more recent studies highlight marked heterogeneity, including patients with preserved ejection fraction but abnormal myocardial strain, isolated right ventricular dysfunction, or reversible diastolic dysfunction [3]. Accordingly, repeated bedside echocardiography, including strain imaging when available, is increasingly recommended for a more accurate evaluation of septic myocardial dysfunction [12].

### 2.2. Mechanisms of Myocardial Dysfunction in Sepsis

**Figure 1:**
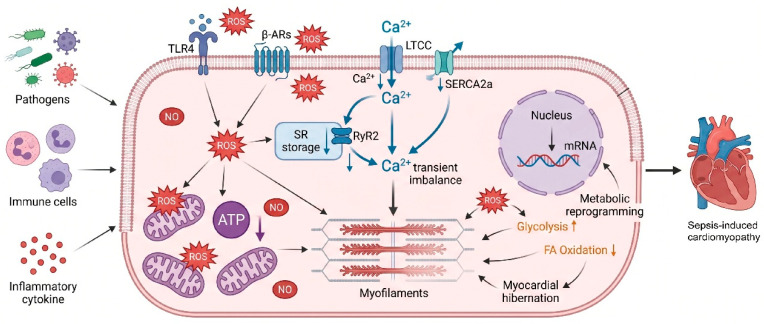
Schematic overview of the major pathophysiological mechanisms underlying sepsis-induced cardiomyopathy (SICM). During sepsis, pathogens, activated immune cells, and circulating inflammatory cytokines trigger myocardial injury through multiple interconnected pathways. Pathogen and inflammation associated signaling activates receptors such as Toll-like receptor 4 (TLR4) and impairs β-adrenergic receptor (β-AR) responsiveness, leading to excessive production of reactive oxygen species (ROS) and nitric oxide (NO). These mediators contribute to mitochondrial dysfunction, reduced ATP generation, and oxidative or nitrosative stress. At the same time, abnormalities in calcium handling, including altered L-type calcium channel (LTCC) activity, impaired sarcoplasmic reticulum (SR) calcium storage, dysregulated ryanodine receptor 2 (RyR2) function, and reduced SERCA2a mediated calcium reuptake, result in calcium transient imbalance and impaired excitation–contraction coupling. Oxidative stress and calcium dysregulation further disrupt myofilament function. In parallel, septic stress induces transcriptional and metabolic reprogramming, characterized by enhanced glycolysis, reduced fatty acid oxidation, and a hibernation like myocardial state. Together, these processes converge to impair myocardial contractility and contribute to the development of SICM.

The pathophysiology of SICM is complex and multifactorial, involving the interplay of systemic inflammation, circulating myocardial depressant factors, endothelial and microcirculatory dysfunction, mitochondrial injury, oxidative and nitrosative stress, calcium-handling abnormalities, autonomic and adrenergic dysregulation, metabolic reprogramming, and iatrogenic factors [13,14] (Figure 1). Recent reviews further stress that SICM cannot be explained by a single pathway, because septic myocardial dysfunction reflects the combined effects of direct cardiomyocyte injury, vascular abnormalities, altered loading conditions, and broader host responses [15]. In addition, newer work suggests that these processes may include dynamic cellular adaptation rather than irreversible injury alone [16]. In particular, recent single-nucleus transcriptomic analysis indicates that during the early phase of sepsis, cardiomyocytes may shift from a contractile phenotype to an injury-responsive phenotype, a process associated with impaired cardiac function but potentially representing an adaptive response to inflammatory and oxidative stress [14].

#### 2.2.1. Inflammatory Responses and Cytokine Storm

Excessive inflammatory activation is a central driver of SICM [17]. During sepsis, pathogen-associated and damage-associated molecular patterns activate pattern-recognition receptors and downstream signaling pathways such as MyD88 [18], NF-κB [19], JNK [20], ERK1/2 [21], and p38 MAPK [22], thereby promoting the release of multiple inflammatory mediators, including TNF-α, IL-1, IL-6, chemokines, adhesion molecules, eicosanoids, and reactive oxygen and nitrogen species [23]. These mediators can impair cardiomyocyte contractility directly and indirectly, alter adrenergic responsiveness, amplify oxidative stress, and disrupt excitation-contraction coupling. Earlier work identified several candidate “myocardial depressant factors,” including TNF-α, IL-1, IL-6, complement-related mediators, and LPS, although more recent clinical observations suggest that circulating cytokine concentrations alone do not consistently correlate with the degree of systolic or diastolic dysfunction, indicating that inflammatory injury in SICM is mechanistically broader than a simple cytokine-dose effect [24]. Thus, inflammation in SICM is now better understood as a network process that drives myocardial dysfunction through both circulating mediators and intracellular stress signaling.

#### 2.2.2. Endothelial Dysfunction and Vascular Leakage

Endothelial injury and microvascular dysfunction are also major contributors to SICM. Sepsis disrupts endothelial barrier integrity, increases vascular permeability, promotes capillary leak, and alters vasomotor tone, which together lead to interstitial edema, abnormal blood flow distribution, and impaired oxygen delivery at the tissue level [25,26,27]. In the coronary microcirculation, these changes may not produce classic epicardial ischemia, because coronary blood flow after resuscitation is often preserved or even increased, but they can still compromise myocardial efficiency through heterogeneous microvascular flow, endothelial edema, fibrin deposition, and inflammatory cell infiltration [28,29]. Recent discussions also highlight the role of endothelial glycocalyx shedding as a contributor to vascular leak, coagulation abnormalities, inflammation, and myocardial edema [30]. In addition, extrinsic hemodynamic factors such as vasodilatation, reduced vascular tone, decreased catecholamine responsiveness, and abnormal afterload conditions further complicate cardiac performance during septic shock [31]. Therefore, SICM should be interpreted not only as intrinsic myocardial failure but also as a disorder of the heart–vasculature–microcirculation unit.

#### 2.2.3. Mitochondrial Dysfunction and Impaired Energetics

Mitochondrial dysfunction is increasingly recognized as a core mechanism of septic myocardial injury [3,32]. In sepsis, inflammatory mediators, reactive oxygen and nitrogen species, and disturbed substrate utilization impair oxidative phosphorylation, alter redox balance, increase mitochondrial uncoupling, reduce membrane potential, and diminish ATP production, while simultaneously promoting excess ROS generation [33]. These abnormalities are accompanied by changes in mitochondrial morphology, calcium homeostasis, fission-fusion dynamics, mitophagy, and biogenesis, all of which can impair the energetic reserve of cardiomyocytes. Because the heart is highly dependent on uninterrupted ATP generation, such defects rapidly translate into reduced contractility and impaired relaxation [34]. At the same time, several reviews propose that SICM may involve a hibernation-like or adaptive metabolic reprogramming state, in which myocardial oxidative activity and contractile work are downregulated to reduce ROS production and prevent irreversible cell injury [35,36,37]. This concept is now supported by recent single-cell level evidence showing that during early sepsis, contractile cardiomyocytes can convert to an injury-responsive subtype characterized by lower contractile and metabolic gene expression; this transition is driven by reduced ERRγ and appears to decrease ROS-associated injury, albeit at the expense of cardiac function [14].

### 2.3. Interaction Between Sepsis and Epigenetic Modifications

**Figure 2:**
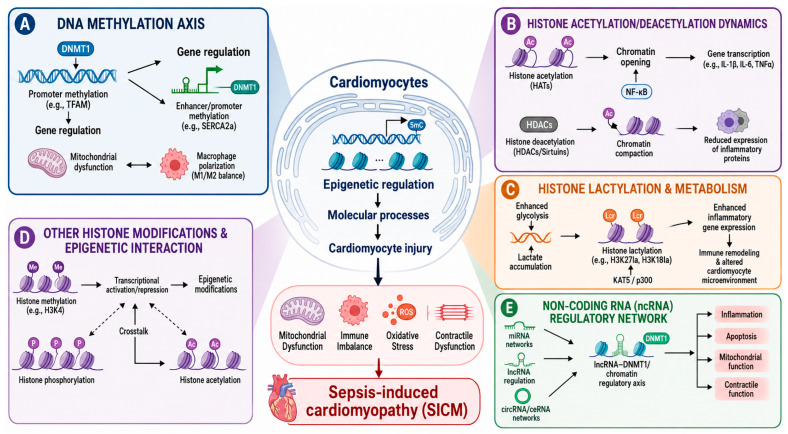
Epigenetic regulatory landscape in sepsis-induced cardiomyopathy (SICM). Schematic overview of the major epigenetic mechanisms involved in SICM pathogenesis. (**A**) DNA methylation: DNMT-mediated methylation regulates mitochondrial function, inflammatory signaling, and cardiomyocyte injury through modulation of target genes such as TFAM and calcium-handling genes. (**B**) Histone acetylation/deacetylation: HATs, HDACs, and sirtuins regulate chromatin accessibility and transcriptional responses associated with inflammation and cellular stress. (**C**) Histone lactylation and metabolism: Sepsis-induced metabolic reprogramming and lactate accumulation promote histone lactylation (e.g., H3K27la and H3K18la), linking metabolic alterations to transcriptional regulation and immune remodeling. (**D**) Other histone modifications: Additional histone marks, including histone methylation, contribute to dynamic chromatin regulation and interact with other epigenetic processes. (**E**) Non-coding RNA networks: miRNAs, lncRNAs, and circRNAs regulate inflammatory responses, apoptosis, mitochondrial dysfunction, and cardiomyocyte injury through RNA-mediated regulatory mechanisms. Collectively, these interconnected epigenetic pathways converge on inflammation, oxidative stress, mitochondrial dysfunction, immune imbalance, and impaired cardiac function, contributing to SICM progression.

Emerging evidence suggests that the septic microenvironment does not merely induce transient hemodynamic and inflammatory changes, but also reshapes gene regulation through epigenetic mechanisms [11]. Severe inflammation, oxidative stress, hypoxia, mitochondrial injury, altered calcium signaling, and metabolic disturbance can modulate DNA methylation, histone modifications, chromatin accessibility, and non-coding RNA expression, thereby altering transcriptional programs in immune cells, endothelial cells, and cardiomyocytes [38] (Figure 2). The latest SICM study further indicates that the pathophysiology of septic myocardial dysfunction likely involves not only biochemical injury but also structured transcriptional reprogramming at the cardiomyocyte level [39].

## 3. Epigenetic Mechanisms in Sepsis-Induced Cardiomyopathy

### 3.1. DNA Methylation in Sepsis-Induced Myocardial Dysfunction

DNA methylation is one of the earliest and most extensively studied epigenetic mechanisms, and it generally regulates gene transcription through CpG methylation within promoter or regulatory regions [40]. Its dynamic balance is maintained by DNA methyltransferases (DNMTs) and demethylation-related mechanisms, and abnormal methylation patterns have been increasingly implicated in sepsis, immune dysregulation, and organ injury [41,42,43]. In SICM, current evidence suggests that DNA methylation is not merely a background epigenetic feature [44,45], but may participate directly in mitochondrial dysfunction, inflammatory amplification, calcium-handling abnormalities, and immune–cardiac crosstalk [4,34,46,47,48,49,50]. However, compared with non-coding RNAs and, more recently, histone lactylation, direct cardiac evidence for DNA methylation in SICM is still developing rather than fully mature.

One of the clearest mechanistic links between DNA methylation and septic myocardial dysfunction comes from work on DNMT1 [51]. In this study, DNMT1 was upregulated in LPS-induced septic myocardial injury, and pharmacologic DNMT1 inhibition with decitabine improved survival and cardiac function, reduced cardiomyocyte apoptosis, promoted macrophage M2 polarization, and attenuated activation of the cGAS–STING pathway. Mechanistically, DNMT1 depletion reduced TFAM promoter methylation, restored TFAM expression, alleviated mitochondrial dysfunction, and limited mitochondrial DNA escape into the cytosol, thereby reducing inflammatory signaling in macrophages and secondarily protecting cardiomyocytes. This study is particularly important because it links DNA methylation not only to transcriptional repression, but also to mitochondrial integrity and immune-cell programming in the septic heart [52,53]. Another informative line of evidence concerns promoter methylation of calcium-handling genes [54,55,56]. Earlier work in cardiomyocytes showed that TNF-α reduced SERCA2a expression by increasing methylation within the SERCA2a promoter and upregulating DNMT1 [57,58]; importantly, this effect was attenuated by the methylation inhibitor 5-aza-2′-deoxycytidine. Although this study was not designed specifically as a modern SICM epigenetics investigation, its relevance to septic myocardial dysfunction is substantial because impaired SERCA2a expression and disturbed Ca2+ reuptake are central to sepsis-related abnormalities in contractility and relaxation [59]. Thus, inflammatory cytokine-driven promoter methylation may represent one route through which sepsis reshapes excitation–contraction coupling in the myocardium. DNA methylation in SICM is also increasingly connected to ncRNA-guided chromatin regulation [60]. A representative example is lncRNA SNHG1, which was shown to promote sepsis-induced myocardial injury by interacting with DNMT1, enhancing DNMT1 enrichment on the Bcl-2 promoter, increasing Bcl-2 promoter methylation, and suppressing Bcl-2 expression. Functionally, SNHG1 knockdown alleviated myocardial inflammation, apoptosis, fibrosis, and dysfunction in LPS-treated mice [50]. This study is notable because it demonstrates that methylation changes in SICM may be directed by upstream RNA scaffolds rather than occurring as isolated enzymatic events. Taken together, current evidence supports the view that DNA methylation in SICM operates at the intersection of mitochondrial injury, immune signaling, apoptosis, and transcriptional repression, and may therefore provide both mechanistic insight and potential therapeutic opportunities.

### 3.2. Histone Acetylation and Deacetylation in SICM

Histone acetylation and deacetylation regulate chromatin accessibility and gene transcription through the opposing actions of histone acetyltransferases (HATs), histone deacetylases (HDACs), and sirtuins [59,60,61]. In the broader field of sepsis biology, acetylation has emerged as a major regulatory layer affecting not only histones but also a wide range of non-histone proteins involved in inflammation, oxidative stress, endothelial permeability, metabolism, and apoptosis [62,63,64,65]. By contrast, direct histone acetylation studies focused specifically on SICM remain relatively limited. Therefore, the current understanding of acetylation in SICM largely relies on integrating general sepsis-associated acetylation biology with known mechanisms of septic myocardial injury. Available sepsis data indicate that histone acetylation can modulate inflammatory gene transcription in a promoter- and cell-specific manner [66]. For example, altered H3 and H4 acetylation in macrophages has been linked to regulation of pro-inflammatory transcription, endotoxin tolerance, and neutrophil recruitment [67]. In parallel, acetylation of non-histone proteins such as NF-κB p65 and HMGB1 has been shown to amplify inflammatory signaling, promote HMGB1 nuclear export and extracellular release, and increase endothelial permeability, all of which are highly relevant to the pathophysiology of septic organ dysfunction [68,69,70,71,72]. Because inflammation, endothelial leak, and microvascular injury are central components of SICM, these acetylation-dependent pathways are likely to influence the myocardial phenotype even when direct cardiac chromatin data are still sparse. Acetylation also intersects closely with metabolic reprogramming and mitochondrial homeostasis, which are highly relevant to SICM [73,74]. Sepsis-related studies have shown that acetylation affects glycolytic and oxidative pathways, and that sirtuin-dependent deacetylation can mitigate oxidative stress and restore metabolic balance. For instance, SIRT3-dependent deacetylation of SDHA dampens aerobic glycolysis in sepsis models, whereas acetylation of mitochondrial proteins in other septic organ injuries aggravates ROS production, mitochondrial dysfunction, and apoptosis [75,76]. Although these observations are not yet specific to septic cardiomyopathy, they provide a strong conceptual basis for considering acetylation/deacetylation as an upstream regulator of the myocardial metabolic and inflammatory disturbances that characterize SICM. Accordingly, HATs, HDACs, and sirtuin-related pathways should be considered plausible regulators of septic inflammatory and metabolic remodeling. However, most available evidence is derived from general sepsis models rather than heart-specific studies, and further investigations are required to clarify their direct roles in SICM [50,77].

### 3.3. Histone Lactylation in SICM

Histone lactylation has rapidly emerged as one of the most important epigenetic mechanisms linking metabolic reprogramming to transcriptional remodeling in sepsis [77,78]. Because lactate accumulation is a hallmark of septic stress and is closely associated with disease severity, lactylation provides a highly relevant framework for understanding how altered metabolism is translated into inflammatory and injury-related gene programs [79,80]. Recent sepsis reviews emphasize that lactate and lactylation form a feedback loop in which metabolic shifts affect chromatin states, which in turn reshape immune and tissue responses [81]. In contrast to the still limited direct evidence for acetylation in SICM, histone lactylation now has several studies directly focused on septic myocardial dysfunction or injury [82]. A major recent advance is the identification of site-specific lactylation events in septic cardiac injury. In recent study, eupatilin ameliorated sepsis-induced myocardial injury by targeting the KAT5–H3K27la axis [83]. The investigators showed that under septic conditions, H3K27la was prominently increased, KAT5 functioned as a critical regulator of this process, and eupatilin reduced inflammatory cytokine expression, improved ejection fraction and fractional shortening, and suppressed H3K27la enrichment at promoters of inflammatory genes. Importantly, the study suggested that in the septic inflammatory microenvironment, KAT5 may act as a context-dependent histone lactylation regulator rather than being restricted to its classical acetyltransferase role, thereby broadening the mechanistic repertoire of lactylation in cardiac sepsis biology. Another study further demonstrated that jaceosidin attenuated sepsis-induced myocardial injury by promoting SIRT2-mediated inhibition of histone H3K18 lactylation [84,85]. In this work, TNF-α-stimulated AC16 cardiomyocytes and LPS-treated mice exhibited increased H3K18la levels, whereas jaceosidin restored SIRT2 expression, reduced H3K18la enrichment, improved cardiac function, and alleviated inflammatory and apoptotic responses. ChIP-PCR further revealed altered H3K18la enrichment at inflammation- and apoptosis-related genes, including IL-6, BAX, and BCL-2, suggesting that SIRT2-mediated regulation of histone lactylation represents a potential metabolic–epigenetic mechanism in SICM. Notably, the biological consequences of lactylation in SICM are not uniformly deleterious and appear to depend on cell type and timing. A study showed that exercise-induced H3K18la in monocyte-derived macrophages, mediated by p300 and counterbalanced by HDAC2, restored cardiac immune homeostasis and preserved cardiac function in SICM [35]. In this model, lactate-educated monocytes promoted an iNOS+Arg1+ macrophage phenotype with both inflammatory and reparative features, and adoptive transfer of highly lactylated monocytes improved cardiac function [86]. This study introduces an important nuance: while cardiomyocyte-associated lactylation may exacerbate inflammatory and apoptotic damage, macrophage-associated lactylation may facilitate immune adaptation and recovery. Therefore, histone lactylation in SICM should be understood as a context-dependent metabolic–epigenetic program rather than a uniformly harmful modification.

### 3.4. Other Histone Modifications and Histone Crosstalk in SICM

Compared with DNA methylation, lactylation, and ncRNAs, other histone modifications remain much less well defined in SICM. Nevertheless, available evidence from sepsis and inflammatory immunology indicates that histone methylation is likely to influence the intensity and duration of inflammatory signaling relevant to septic cardiac injury [87]. A representative example is Ash1l, a histone H3K4 methyltransferase that suppresses IL-6 and TNF production in TLR-triggered macrophages by inducing A20 expression, thereby restraining NF-κB and MAPK signaling and protecting mice from sepsis [88]. Although this mechanism has not been directly mapped onto cardiomyocytes in SICM, it provides a strong mechanistic rationale for considering histone methylation as an upstream determinant of the inflammatory environment to which the septic heart is exposed. Recent SICM-related lactylation studies suggest that acetylation and lactylation share overlapping enzymatic logic and, potentially, overlapping lysine-centered chromatin landscapes. In the exercise-preconditioning model of SICM, p300 and HDAC2 were identified as functionally relevant writer/eraser components for H3K18la, whereas in cardiomyocyte-focused models SIRT2 selectively suppressed H3K18la with minimal disruption of the broader acetylation network [35]. In addition, the KAT5–H3K27la study suggests that classical acetyltransferase-family enzymes may act as context-dependent lactylation regulators under septic stress. These findings support the idea that chromatin remodeling in SICM is not organized as separate “acetylation”, “lactylation”, or “methylation” silos, but rather as an integrated network of interacting histone marks. Direct evidence for phosphorylation, ubiquitination, or other histone PTMs in SICM is still scarce, and this remains a major area for future research.

### 3.5. Non-Coding RNAs (ncRNAs) in Sepsis-Induced Cardiomyopathy

Among the epigenetic regulators studied in SICM, non-coding RNAs are currently the most extensively investigated. These include microRNAs, long non-coding RNAs (lncRNAs), and circular RNAs (circRNAs), which together regulate inflammatory signaling, oxidative stress, apoptosis, calcium handling, mitochondrial function, and immune-cell communication (Table 1). A systematic review of sepsis-induced cardiac dysfunction summarized at least 77 miRNAs implicated in this process, while later reviews have expanded the field to include lncRNA- and circRNA-based regulatory networks [89]. Overall, ncRNAs appear to form a multilayered post-transcriptional and epigenetic control system in SICM, although the level of evidence varies considerably across RNA classes.

#### 3.5.1. MicroRNAs in Inflammation, Apoptosis, and Contractile Dysfunction of SICM

MicroRNAs are the best-characterized ncRNAs in SICM. A large body of work indicates that several miRNAs regulate inflammatory, oxidative, apoptotic, and contractile pathways in septic myocardium, often through NF-κB-centered signaling [89,90,91]. Protective miRNAs include miR-146a, whose upregulation attenuates myocardial dysfunction and inflammation [92]; miR-125b, whose restoration improves cardiac function [93]; and miR-223, whose deficiency enhances myocardial inflammation [94]. By contrast, miR-21–3p and miR-155 are often reported to be upregulated in septic myocardium, and in some studies their inhibition attenuated dysfunction and inflammation [95,96]. Additional miRNAs such as miR-124a, miR-135a, and miR-214 have also been linked to myocardial inflammation, apoptosis, and functional decline [97,98,99]. At the same time, available data also show that some miRNAs, especially miR-155, may have context-dependent and even apparently opposing effects depending on model, timing, and downstream target selection [100]. Thus, miRNA biology in SICM is extensive but not always directionally uniform. More recent sepsis reviews further suggest that circulating and tissue miRNA signatures may provide additional biological and clinical insight. Cardiac-associated miRNAs such as miR-208a [101], miR-133a [102], and miR-499 [103] have been reported to rise in animal models and patients with sepsis-associated cardiac dysfunction, while lower levels of miR-146a [104] and miR-223 [105] have been associated with worse organ outcomes and possibly a more immunosuppressive profile. Therefore, miRNAs in SICM should be viewed not only as mechanistic effectors, but also as candidate biomarkers reflecting injury burden, immune phase, and organ specificity.

#### 3.5.2. Long Non-Coding RNAs in Mitochondrial Dysfunction and Cardiomyocyte Injury

Long non-coding RNAs are increasingly recognized as important regulators of mitochondrial dysfunction and cardiomyocyte injury in SICM [106]. The existing literature indicates that many lncRNAs participate in mitochondrial membrane potential regulation, ROS production, ATP metabolism, and mitochondrial apoptosis. For example, RMRP protects against mitochondrial dysfunction and cardiomyocyte apoptosis through the miR-1–5p/HSPA4 axis [107]; H19 suppresses inflammatory cytokine production and mitochondrial apoptosis through the miR-93–5p/SORBS2 axis [108,109]. Other studies suggest that Xist is associated with reduced PGC-1α expression and decreased ATP production, thereby linking lncRNA dysregulation to impaired bioenergetic recovery [110]. These findings support the idea that lncRNAs are highly relevant to the mitochondrial dimension of SICM pathogenesis. Beyond mitochondria, lncRNAs also modulate cardiomyocyte injury more broadly through inflammatory and apoptotic pathways. MIAT has been shown to promote oxidative stress and inflammation through the miR-330–5p/TRAF6/NF-κB axis, while MALAT1 enhances myocardial inflammation through miR-125b- and miR-150–5p-related pathways [111]. NEAT1 promotes myocardial cell injury by suppressing miR-144–3p [112], and other lncRNAs such as CRNDE, CYTOR, and KCNQ1OT1 have been linked to apoptosis-related signaling involving SIRT1 or XIAP [113]. Taken together, the lncRNA landscape in SICM points to a network-level regulatory layer that integrates mitochondrial injury with inflammatory signaling and cell fate determination.

#### 3.5.3. lncRNA-Directed Epigenetic Regulation in SICM

##### lncRNA–miRNA Axes

A major mechanism of lncRNA function in SICM is the competing endogenous RNA (ceRNA) model, whereby lncRNAs sponge specific miRNAs and thereby derepress downstream targets. Representative examples include MALAT1–miR-125b/p38 MAPK–NF-κB [105], MALAT1–miR-150–5p/NF-κB [106], MIAT–miR-330–5p/TRAF6 [114], RMRP–miR-1–5p/HSPA4 [115], H19–miR-93–5p/SORBS2 [60], CYTOR–miR-24/XIAP [116], and KCNQ1OT1–miR-192–5p/XIAP [117]. These axes regulate apoptosis, oxidative stress, inflammatory signaling, mitochondrial integrity, and ultimately cardiac performance. The ceRNA model is therefore highly useful for organizing lncRNA biology in SICM, especially because it explains how a single lncRNA can coordinate multiple downstream phenotypes through a shared signaling hub.

##### lncRNA–DNMT1/Chromatin Regulator Axes

Importantly, not all lncRNAs in SICM act only through miRNA sponging. Some directly interface with chromatin regulators and DNA methylation machinery. The best current example is SNHG1, which physically interacts with DNMT1, promotes DNMT1 occupancy at the Bcl-2 promoter, increases promoter methylation, suppresses Bcl-2 expression, and thereby aggravates inflammation, apoptosis, and myocardial dysfunction in septic models [118,119,120,121]. This is one of the strongest current demonstrations that lncRNAs in SICM can function as bona fide epigenetic organizers rather than merely post-transcriptional sponges. As more chromatin-centered lncRNA studies emerge, this category is likely to become increasingly important for understanding how stable yet reversible transcriptional changes are established in septic myocardium.

#### 3.5.4. Diagnostic and Therapeutic Potential of ncRNA Networks in SICM

The translational appeal of ncRNA biology in SICM lies in the fact that many of these molecules are measurable in blood, display relative tissue or pathway specificity, and directly regulate clinically relevant mechanisms such as inflammation, mitochondrial dysfunction, calcium dysregulation, apoptosis, and immune-cell polarization. For instance, circulating miR-21–3p has been reported to be higher in septic patients with cardiac dysfunction than in septic patients without cardiac involvement [88], while cardiac-associated miRNAs such as miR-208a [122], miR-133a [123], and miR-499 [124] may help reflect myocardial injury burden. Likewise, the remarkable stability of circRNAs and the availability of exosome-associated ncRNAs make them promising candidates for biomarker development and intercellular signaling analysis in sepsis-associated cardiac injury. From a therapeutic perspective, miRNA mimics, antagomiRs, lncRNA silencing, and exosome-based RNA delivery all appear promising in preclinical models. However, several major limitations remain: ncRNA effects are often time-dependent and cell-type specific; the same RNA can exert divergent functions across disease stages; systemic delivery raises issues of off-target effects and pharmacokinetics; and current evidence is still dominated by cell and animal studies rather than prospective clinical validation [52,116]. Therefore, while ncRNA networks represent the most advanced epigenetic area in SICM from a mechanistic standpoint, their clinical translation will require better phenotyping of patients, improved delivery platforms, and deeper understanding of stage-specific RNA programs in septic myocardial dysfunction.

## 4. Interplay Between Inflammation and Epigenetic Changes in SICM

### 4.1. Inflammatory Pathways Involved in Epigenetic Modulation

Inflammation is not only a central pathological driver of SICM, but also a major upstream regulator of epigenetic remodeling. During sepsis, pathogen-associated and damage-associated molecular patterns activate Toll-like receptor signaling, NF-κB, JAK/STAT, MAPK, and inflammasome-related pathways, which in turn influence the activity of DNA methyltransferases, histone-modifying enzymes, and multiple classes of non-coding RNAs [54]. Through these mechanisms, acute inflammatory stress can be translated into broader transcriptional reprogramming in cardiomyocytes, endothelial cells, and immune cells, thereby promoting persistent myocardial dysfunction even when the initial infectious trigger is controlled.

### 4.2. Epigenetic Control of Cytokine Production and Immune Response

Epigenetic mechanisms also regulate the production of cytokines and the overall immune response during sepsis. DNA methylation and histone modifications can alter the accessibility of promoters and enhancers of inflammatory genes, while microRNAs, lncRNAs, and circRNAs fine-tune post-transcriptional signaling networks involved in TNF-α, IL-1β, IL-6, and other mediators [124]. In this way, epigenetic regulation may determine whether inflammatory signaling is transient and adaptive or prolonged and maladaptive. In SICM, such regulation is particularly important because excessive or unresolved cytokine signaling contributes directly to myocardial depression, impaired excitation-contraction coupling, mitochondrial dysfunction, and cardiomyocyte death [125,126,127,128,129,130]. Therefore, the inflammatory phenotype of the septic heart should be understood not only as a consequence of infection, but also as a product of dynamic epigenetic control.

### 4.3. The Epigenetic Influence of Oxidative Stress on Myocardial Cells

Oxidative and nitrosative stress are closely intertwined with inflammation in sepsis and represent another important route through which the septic microenvironment affects epigenetic regulation. Reactive oxygen and nitrogen species can modify the activity of chromatin-modifying enzymes, disturb methyl donor availability, alter histone marks, and reshape non-coding RNA expression, thereby influencing gene programs related to mitochondrial quality control, apoptosis, autophagy, and contractile function [64,131,132,133,134,135,136,137]. In myocardial cells, this creates a feed-forward cycle in which oxidative injury promotes epigenetic dysregulation, and epigenetic dysregulation further weakens antioxidant defenses and cellular adaptation [138]. As a result, oxidative stress in SICM should not be viewed solely as a biochemical injury mechanism, but also as an important contributor to sustained transcriptional and phenotypic remodeling in the septic heart [139].

## 5. Therapeutic Targets in Sepsis-Induced Cardiomyopathy: From Epigenetic Mechanisms to Clinical Application

As discussed in the previous sections, multiple epigenetic regulators have emerged as important contributors to the pathogenesis of sepsis-induced cardiomyopathy (SICM), making them attractive candidates for therapeutic intervention. Current evidence indicates that DNA methylation, histone acetylation/deacetylation, histone lactylation, and non-coding RNA networks participate in the regulation of inflammatory activation, mitochondrial dysfunction, cardiomyocyte apoptosis, immune-cell polarization, and metabolic reprogramming in septic cardiac injury [57,95]. Among these mechanisms, several targets have already shown direct cardioprotective effects in preclinical models of septic myocardial injury and cardiac dysfunction. For example, inhibition of DNMT1 by decitabine improved survival and cardiac function, attenuated cardiomyocyte apoptosis, and suppressed mtDNA-mediated cGAS–STING activation by restoring TFAM expression [139]. More recent studies further identified lactylation-related targets with therapeutic promise: eupatilin alleviated septic myocardial injury by targeting the KAT5–H3K27la axis, whereas jaceosidin improved cardiac function by promoting SIRT2-mediated inhibition of H3K18 lactylation [45,57]. In addition, adoptive transfer of highly histone-lactylated monocytes restored cardiac function in experimental SICM, suggesting that metabolic–epigenetic reprogramming of cardiac immune cells may also be therapeutically exploitable. Beyond these more direct examples, accumulating sepsis literature suggests that HDACs, sirtuins, and ncRNA networks may also represent promising intervention points, although cardiac-specific translational evidence remains less mature [2,11]. Accordingly, Table 1 summarizes the principal epigenetic targets currently implicated in SICM, their main functions, and the potential therapeutic strategies supported by current preclinical evidence.

Although epigenetic-targeting strategies have shown promising protective effects in experimental models, several challenges must be addressed before their clinical translation into SICM management. First, safety remains a major concern because epigenetic regulators frequently control broad transcriptional programs across multiple tissues. For example, decitabine-mediated DNMT inhibition may influence not only cardiac pathways but also immune responses, hematopoiesis, and genomic stability, raising concerns regarding systemic toxicity and optimal therapeutic windows in critically ill septic patients.

Second, potential off-target effects represent another important limitation. Unlike conventional drug targets that often regulate specific signaling pathways, epigenetic enzymes such as DNMTs, HDACs, and sirtuins participate in diverse cellular processes. Therefore, broad modulation of these regulators may induce unintended transcriptional alterations or affect host immune responses, which are particularly relevant in sepsis where immune balance is highly dynamic.

Third, effective delivery remains a major challenge. Although compounds such as eupatilin and jaceosidin have demonstrated beneficial effects in experimental models, their pharmacokinetic properties, tissue distribution, myocardial accumulation, and long-term safety profiles remain insufficiently characterized. Developing cardiac-targeted delivery systems or identifying patient-specific molecular signatures may help improve therapeutic specificity and reduce systemic adverse effects.

Importantly, current evidence supporting epigenetic-based therapies in SICM is almost exclusively derived from in vitro and animal studies. No epigenetic-modifying therapy has yet been clinically validated for SICM, and available human evidence remains largely limited to observational biomarker studies. Future clinical translation will require well-designed studies integrating molecular profiling, standardized SICM phenotyping, and long-term safety assessment to determine whether epigenetic interventions can provide effective and clinically applicable therapeutic strategies.

## 6. Current Limitations and Future Perspectives of Epigenetic Research in SICM

Importantly, the absence of universally accepted diagnostic criteria for SICM remains a fundamental limitation. Variations in definitions based on left ventricular ejection fraction, strain imaging, right ventricular function, and septic shock severity may lead to heterogeneous patient classification and complicate the interpretation of epigenetic signatures and biomarker studies (Supplementary Table S1).

Although increasing evidence supports the involvement of epigenetic regulation in SICM, the strength of evidence differs among regulatory mechanisms. DNA methylation studies, particularly DNMT1-mediated regulation of mitochondrial function and inflammatory signaling, provide important mechanistic insights; however, most findings remain limited to experimental models with insufficient human validation. Similarly, histone modifications, including acetylation, deacetylation, and lactylation, have revealed important links between metabolic stress and transcriptional remodeling, but their roles are mainly supported by preclinical studies. Histone lactylation represents an emerging area, with studies identifying functional roles for H3K27la and H3K18la; however, the temporal dynamics, cell-specific regulation, and causal versus adaptive nature of these modifications remain unclear.

Non-coding RNAs, particularly microRNAs and lncRNAs, are among the most extensively studied epigenetic regulators in SICM. Nevertheless, current evidence largely relies on gain- or loss-of-function experiments, and the clinical relevance of circulating RNA signatures requires further validation. Moreover, the complex interactions among ncRNAs, epigenetic enzymes, and downstream targets remain incompletely understood.

Several challenges limit the clinical translation of epigenetic findings in SICM. The lack of standardized diagnostic criteria, heterogeneity among experimental models, limited availability of human cardiac samples, and insufficient phenotype-specific molecular characterization complicate the interpretation and translation of current discoveries. In addition, epigenetic therapies face challenges related to tissue specificity, delivery efficiency, reversibility, and potential off-target effects. Future studies integrating single-cell sequencing, single-cell ATAC-seq, spatial transcriptomics, and multi-omics approaches may help define cell-specific epigenetic landscapes and identify clinically relevant biomarkers. Prospective clinical studies are also needed to determine whether epigenetic signatures can improve SICM phenotyping, prognostic assessment, and individualized therapeutic strategies.

## 7. Conclusions and Perspectives

Sepsis-induced cardiomyopathy is a complex, heterogeneous, and potentially reversible form of septic organ dysfunction in which inflammatory injury, microvascular and endothelial disturbance, mitochondrial damage, calcium-handling abnormalities, metabolic reprogramming, and cellular phenotypic adaptation converge to impair myocardial performance. On this background, epigenetic regulation has emerged as a mechanistic layer that helps explain how systemic septic stress is translated into coordinated transcriptional and post-transcriptional remodeling in the heart and its immune microenvironment. The evidence reviewed here indicates that DNA methylation, histone acetylation/deacetylation, histone lactylation, and non-coding RNAs are all involved in SICM pathogenesis, although the strength of evidence differs across these categories. In particular, recent studies have moved the field beyond descriptive association by identifying specific regulatory axes, such as DNMT1–TFAM–mtDNA–cGAS/STING, KAT5–H3K27la, and SIRT2–H3K18la, which connect epigenetic remodeling to mitochondrial dysfunction, inflammatory amplification, cardiomyocyte injury, and cardiac dysfunction in septic models.

At the same time, the therapeutic implications of these findings are increasingly compelling but remain largely preclinical. DNMT1 inhibition with decitabine, suppression of KAT5-dependent H3K27 lactylation by eupatilin, enhancement of SIRT2-mediated inhibition of H3K18 lactylation by jaceosidin, and adoptive transfer of highly histone-lactylated monocytes have all shown cardioprotective effects in experimental septic myocardial injury or dysfunction. In parallel, the expanding literature on miRNAs and lncRNAs suggests that ncRNA networks may serve as both biomarkers and intervention targets for inflammation, apoptosis, mitochondrial dysfunction, and contractile impairment in SICM. However, no epigenetic therapy has yet entered routine clinical management of SICM, and most candidate strategies still face major barriers related to disease heterogeneity, timing of intervention, cell-type specificity, systemic off-target effects, and the lack of prospective validation in human patients.

Future studies should therefore focus on integrating mechanistic epigenetic insights with clinically meaningful SICM phenotyping. This will require more precise definitions of septic cardiac dysfunction, serial imaging and biomarker profiling, and greater incorporation of human tissue-based and high-resolution omics approaches. Single-cell RNA sequencing (scRNA-seq) may help identify cell-specific transcriptional responses and reveal distinct cardiomyocyte, macrophage, endothelial, and immune-cell states associated with different SICM phenotypes. Single-cell ATAC-seq (scATAC-seq) can further define chromatin accessibility landscapes and identify regulatory elements underlying epigenetic remodeling. Spatial transcriptomics provides additional information regarding the anatomical distribution of molecular alterations within the septic heart, allowing investigation of regional interactions among cardiomyocytes, immune cells, and the vascular microenvironment. Moreover, integrated multi-omics approaches combining transcriptomics, epigenomics, proteomics, and metabolomics may facilitate the identification of metabolic–epigenetic networks and phenotype-specific biomarkers. Particular attention should be given to metabolic–epigenetic coupling, especially lactylation-related pathways, RNA-based regulatory networks, and the development of selective delivery systems capable of targeting defined molecular nodes without broadly disrupting host defense. Ultimately, these approaches may enable a transition from syndrome-based classification toward molecularly stratified diagnosis and personalized therapy for SICM.

## Figures and Tables

**Table 1:** Putative epigenetic targets and candidate therapeutic strategies for SICM.

Epigenetic Category	Specific Target(s)	Mechanism	Validation Status	Main Therapeutic Effect Reported	Evidence Level	Model/Population	Therapeutic Readiness	Reference
DNA Methylation	DNMT1	Promotes TFAM promoter methylation, mitochondrial dysfunction, mtDNA cytosolic escape, cGAS-STING activation, macrophage M1 polarization, and cardiomyocyte apoptosis	DNMT1 inhibition (e.g., decitabine, si-DNMT1)	Aggravating	In vitro + Animal	LPS-induced cardiomyocyte/macrophage models and septic mouse models	Preclinical candidate	[57]
Histone Acetylation/Deacetylation	HDACs/sirtuins	Regulate inflammatory transcription, oxidative stress, endothelial permeability, and metabolic dysfunction in sepsis-associated organ injury, with likely relevance to SICM	HDAC inhibition/sirtuin modulation	Ameliorative	In vitro + Animal	Experimental sepsis models	Experimental/exploratory	[83,84]
	KAT5-H3K27la axis	Promotes H3K27 lactylation and inflammatory gene activation under septic stress	KAT5/H3K27la suppression (e.g., eupatilin)	Aggravating	In vitro + Animal	LPS-induced cardiomyocyte models and septic mouse models	Preclinical candidate	[89,90]
	SIRT2-H3K18la axis	H3K18la drives inflammatory and apoptotic gene transcription, including IL-6, BAX, and BCL-2-related signaling	SIRT2 activation/H3K18la inhibition (e.g., jaceosidin)	Ameliorative	In vitro + Animal	TNF-α-stimulated AC16 cardiomyocytes and LPS-induced septic mouse models	Preclinical candidate	[90,91]
	H3K18la in monocyte-derived macrophages	Reprograms cardiac macrophages toward immune homeostasis and reparative function in SICM	Exercise-induced metabolic priming/adoptive transfer of highly lactylated monocytes	Ameliorative	In vitro + Animal	Septic mouse models and monocyte-derived macrophage models	Preclinical intervention	[36]
MicroRNAs	miR-146a, miR-125b, miR-223, miR-21–3p, miR-155, miR-23b, miR-124a	Generally protective; restoration suppresses inflammation, apoptosis, and myocardial dysfunction	miRNA restoration (mimics/agomiRs)	Ameliorative	In vitro + Animal	Polymicrobial sepsis and LPS-induced cardiac dysfunction models; cardiomyocyte and exosome-based systems	Exploratory/Preclinical	[92,93,94,95,96,97,98,99,100,101,102]
	miR-21–3p, miR- 135a	Generally pathogenic when elevated; associated with aggravated inflammation and cardiac dysfunction	miRNA inhibition (antagomiRs/silencing)	Aggravating	In vitro + Animal	LPS-induced and polymicrobial sepsis cardiac dysfunction models	Exploratory/Preclinical	[103,104,105,106]
Long Non-Coding RNAs	RMRP, H19, CRNDE, CYTOR, KCNQ1OT1, TUG1	Generally protective; preserve mitochondrial function, reduce ROS, inhibit apoptosis, or restrain inflammation	lncRNA restoration/overexpression/EV-based delivery	Ameliorative	In vitro + Animal	LPS-induced cardiomyocyte models and septic mouse models	Exploratory/Preclinical	[106,107,108,109,110,111,112,113]
	MALAT1, MIAT, Xist, NEAT1, HOTAIR	Generally pro-injury; promote inflammation, oxidative stress, mitochondrial dysfunction, apoptosis, or myocardial dysfunction	lncRNA silencing/knockdown	Aggravating	In vitro + Animal	LPS-induced cardiomyocyte injury models and experimental SICM/sepsis mouse models	Exploratory/Preclinical	[114,115,116,117,118,119,120,121]
lncRNA-Directed Epigenetic Regulation	SNHG1-DNMT1-Bcl-2 axis	SNHG1 recruits DNMT1 to the Bcl-2 promoter, increases promoter methylation, suppresses Bcl-2 expression, and aggravates myocardial injury	SNHG1 silencing or DNMT1-targeted intervention	Aggravating	In vitro + Anima	LPS-induced cardiomyocyte injury models and septic mouse models	Exploratory/Preclinical	[64]
lncRNA-miRNA Axes	MALAT1-miR-125b, MALAT1-miR-150–5p, MIAT-miR-330–5p, RMRP-miR-1–5p, H19-miR-93–5p, CYTOR-miR-24, KCNQ1OT1-miR-192–5p, NEAT1-miR-144–3p, Xist-miR-7a-5p	Coordinate apoptosis, inflammation, mitochondrial dysfunction, autophagy, and cardiomyocyte survival	Axis-specific RNA modulation	Ameliorative	In vitro + Animal	LPS-induced cardiomyocyte injury models and experimental sepsis/SICM mouse models	Exploratory/Preclinical	[117,119,121,122,123,124,125]

## Data Availability

No new data were created or analyzed in this study. Data sharing is not applicable to this article.

## Supplementary Materials

The following supporting information can be downloaded at https://www.mdpi.com/article/10.3390/ijms27156767/s1.

## Abbreviations

SICM	sepsis-induced cardiomyopathy;
SIMD	sepsis-induced myocardial dysfunction;
DNMT	DNA methyltransferase;
HAT	histone acetyltransferase;
HDAC	histone deacetylase;
ncRNA	non-coding RNA;
miRNA	microRNA;
lncRNA	long non-coding RNA;
circRNA	circular RNA;
ROS	reactive oxygen species;
mtDNA	mitochondrial DNA;
cGAS–STING	cyclic GMP–AMP synthase–stimulator of interferon genes;
TFAM	mitochondrial transcription factor A;
KAT5	lysine acetyltransferase 5;
SIRT2	sirtuin 2;
SIRT3	sirtuin 3;
H3K18la	histone H3 lysine 18 lactylation;
H3K27la	histone H3 lysine 27 lactylation;
scRNA-seq	single-cell RNA sequencing;
