# Supplementary material for: Epigenetic Mechanisms in Sepsis-Induced Cardiomyopathy: From Pathophysiology to Therapeutic Targets
Source: Int J Mol Sci. 2026 Jul 28;27(15):6767. doi: 10.3390/ijms27156767 (PMC13466546; doi:10.3390/ijms27156767)
Supplement: Supplementary file 1 [file ijms-27-06767-s001.zip › ijms-4383795-supplementary.pdf]

Supplementary Table 1. Diagnostic heterogeneity of sepsis-induced cardiomyopathy in current studies

Diagnostic approach / phenotype	Definition or commonly used criteria	Advantages	Limitations and implications for SICM research
**Left ventricular ejection fraction (LVEF)-based definition**	Reduced LVEF during sepsis (commonly <40–50%, thresholds vary among studies)	Widely available, clinically established, easy to assess by echocardiography	LVEF is highly load-dependent in septic shock and may normalize rapidly after resuscitation; may underestimate early or subtle myocardial dysfunction
**Global longitudinal strain (GLS)-based assessment**	Reduced myocardial deformation despite preserved or reduced LVEF	More sensitive for detecting subclinical myocardial dysfunction and impaired contractility	Limited availability and lack of standardized cutoff values; strain abnormalities may identify different SICM phenotypes compared with LVEF-based studies
**Left ventricular diastolic dysfunction phenotype**	Impaired relaxation, altered E/e′ ratio, or other diastolic parameters	Reflects abnormalities in myocardial relaxation and ventricular compliance	Diagnostic criteria vary substantially; isolated diastolic dysfunction remains controversial as an independent SICM phenotype
**Right ventricular dysfunction phenotype**	RV systolic impairment assessed by TAPSE, RV fractional area change, or other parameters	Recognizes the contribution of RV failure in severe sepsis and mechanical ventilation	RV function is influenced by pulmonary vascular resistance, ventilation strategies, and volume status; criteria are not uniformly defined
**Biventricular dysfunction phenotype**	Combined LV and RV impairment	Reflects severe myocardial involvement and systemic hemodynamic burden	Often observed in critically ill populations with multiple confounding factors
**Septic shock-based populations**	SICM evaluated in patients with septic shock, vasopressor requirement, or severe organ dysfunction	Represents clinically severe disease populations with high mortality risk	Disease severity, treatment interventions, and timing of assessment may influence cardiac phenotypes and molecular alterations
